# Compress^®^ Knee Arthroplasty Has 80% 10-year Survivorship and Novel Forms of Bone Failure

**DOI:** 10.1007/s11999-012-2635-6

**Published:** 2012-10-09

**Authors:** John H. Healey, Carol D. Morris, Edward A. Athanasian, Patrick J. Boland

**Affiliations:** Orthopaedic Service of the Department of Surgery, affiliated with Weill Medical College of Cornell University, Memorial Sloan-Kettering Cancer Center, 1275 York Avenue, New York, NY 10065 USA

## Abstract

**Background:**

Compliant, self-adjusting compression technology is a novel approach for durable prosthetic fixation of the knee. However, the long-term survival of these constructs is unknown.

**Questions/purposes:**

We therefore determined the survival of the Compress^®^ prosthesis (Biomet Inc, Warsaw, IN, USA) at 5 and 10 actuarial years and identified the failure modes for this form of prosthetic fixation.

**Methods:**

We retrospectively reviewed clinical and radiographic records for all 82 patients who underwent Compress^®^ knee arthroplasty from 1998 to 2008, as well as one patient who received the device elsewhere but was followed at our institution. Prosthesis survivorship and modes of failure were determined. Followup was for a minimum of 12 months or until implant removal (median, 43 months; range, 6–131 months); 28 patients were followed for more than 5 years.

**Results:**

We found a survivorship of 85% at 5 years and 80% at 10 years. Eight patients required prosthetic revision after interface failure due to aseptic loosening alone (n = 3) or aseptic loosening with periprosthetic fracture (n = 5). Additionally, five periprosthetic bone failures occurred that did not require revision: three patients had periprosthetic bone failure without fixation compromise and two exhibited irregular prosthetic osteointegration patterns with concomitant fracture due to mechanical insufficiency.

**Conclusions:**

Compress^®^ prosthetic fixation after distal femoral tumor resection exhibits long-term survivorship. Implant failure was associated with patient nonadherence to the recommended weightbearing proscription or with bone necrosis and fracture. We conclude this is the most durable FDA-approved fixation method for distal femoral megaprostheses.

**Level of Evidence:**

Level IV, therapeutic study. See Instructions for Authors for a complete description of levels of evidence.

**Electronic supplementary material:**

The online version of this article (doi:10.1007/s11999-012-2635-6) contains supplementary material, which is available to authorized users.

## Introduction

Megaprostheses need improved bone fixation to reduce the rate of aseptic loosening associated with stemmed implants. Young patients cured of tumors have a long life expectancy and a compelling need for prosthetic fixation that is equally long-lasting. A recently developed strategy is compliant compressive fixation that uses compression, via a short traction bar, to stimulate osteointegration at the bone-prosthetic interface, promote hypertrophy of the loaded bone, and avoid stress bypass of the host bone around a stiff intramedullary stem [2]. The Compress^®^ Compliant Pre-Stress Implant (Biomet Inc, Warsaw, IN, USA), a rotating-hinge knee prosthesis, was approved by the FDA based on data from an unpublished short-term feasibility study, conducted by the manufacturer, that showed no difference in the acute complication rate and equivalent functional outcome scores compared with a cemented stem coupled to the same rotating-hinge articulation (Orthopaedic Salvage System [OSS™]; Biomet). Published studies of this device include an investigation in 26 patients, among whom only 10 had followup longer than 24 months [4], a study of 26 patients followed for a period of 0.3 to 9.2 years [23], and a study of 41 patients followed for 3 to 97 months [9]. These studies suggest projected 10-year prosthetic survival is at least 80%, but the number of cases is small and the number followed for this duration is miniscule.

We therefore determined the survival of the Compress^®^ prosthesis at 5 and 10 actuarial years and identified the failure modes for this form of prosthetic fixation. Finally, the results were compared with those reported in a comprehensive review of the literature to establish the superiority of this method of fixation compared to those previously reported.

## Patients and Methods

We retrospectively reviewed all 82 patients treated for distal femoral reconstructions after major bone resection from January 1998 to November 2008 at our institution. This implant was used for all primary and secondary (revision) femoral reconstructions, except for cases in which the remaining bone was inadequate due to insufficient cortical thickness, patient age of more than 70 years, metastatic disease, or prior irradiation of the femur (Table 1). Surgery was performed for tumor reconstruction in 80 patients and for noncancer revision TKA with massive bone loss in two patients. Followup was for a minimum of 12 months or until implant removal (mean, 48.4 months; median, 43 months; range, 6–131 months). Twenty-eight patients (> 33%) were followed for longer than 5 years. This study cohort includes the 41 patients in our earlier study who were followed for a mean of 45 months (range, 3–97 months) [9]. One additional patient underwent knee arthroplasty elsewhere and was followed at our institution. This patient was included for illustrative purposes because of an unusual complication (Type IIB bone failure, see below) that helped to establish our classification scheme of periprosthetic bone failure. The patient was not included in our patient cohort total or in the survivorship analysis. Patients were operated on for a variety of cancer diagnoses (Table 2). Our institutional review board approved this study.

**Table 1:** Contraindications for use of the Compress^®^ device for knee arthroplasty

Cortical thickness of less than 2.5 mm
Pre- or postoperative bone irradiation, precluding osteointegration
Extraarticular resection of knee (an articulated implant, such as the Burstein-Lane^®^ implant, would be indicated)
Inadequate or unreconstructable soft tissue envelope (a very low-profile implant, such as the GUEPAR^®^ implant, would be indicated)
Metastatic disease that mandates immediate weightbearing (precludes the requisite 3 months of protected weightbearing)
Inability to cooperate with the postoperative program of early, protected weightbearing

**Table 2:** Patient demographic and clinical characteristics

Characteristic	Value
Number of patients	82
Age (years)*	20.4 (14–63)
Sex (number of patients)	
Male	40
Female	42
Reconstruction surgery (number of patients)	
Primary	64
Revision	18
Tumor diagnosis (number of patients)	
High-grade osteogenic sarcoma	64
Chondrosarcoma	5
Malignant fibrohistiocytoma	5
Giant cell tumor	3
Low-grade osteogenic sarcoma	2
Other tumor	1
No tumor (arthroplasty revision)	2

* The value is expressed as the median, with range in parentheses.

**Fig. 1A–B:**
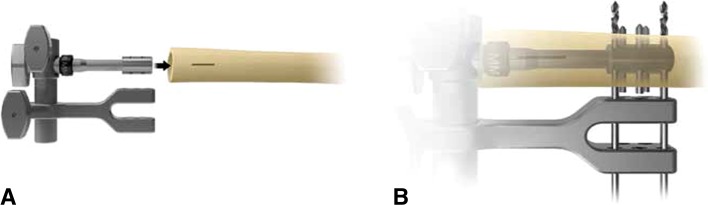
(**A**) A diagram illustrates the outrigger that aligns the external drill guide with the intramedullary anchor plug. (**B**) A diagram demonstrates how the drill bits (through the outer two holes) and ultimately the fixation pins (through the central three holes) align. Reprinted with permission of Biomet Inc from Compress^®^ Compliant Pre-Stress Device Orthopaedic Salvage System: surgical technique. Available at: http://www.biomet.com/orthopedics/getfile.cfm?id=1711&rt=inline. ©2012 Biomet Inc.

**Fig. 2A–B:**
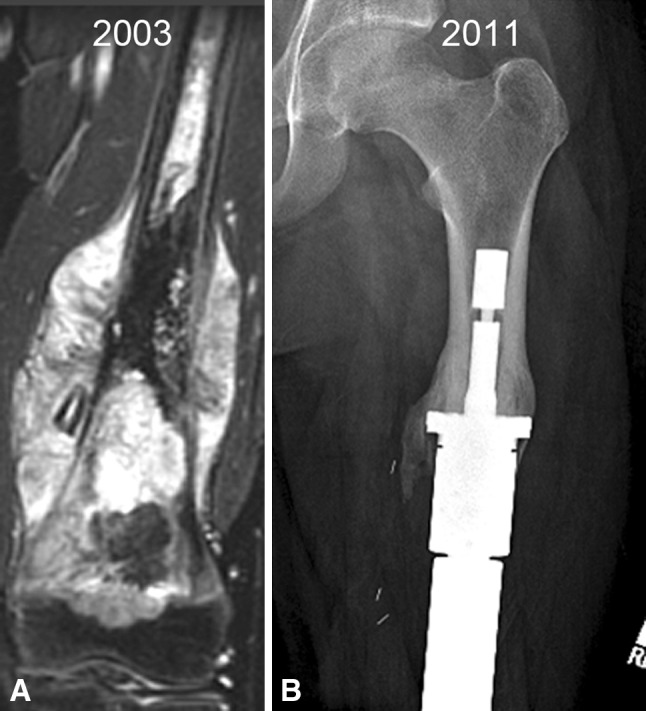
The Compress^®^ is ideally suited for situations where there is a short remaining intramedullary canal that can only accommodate a short stem and fixation would be compromised. (**A**) An image shows the femur of a 15-year-old patient who had resection of a 25-cm osteogenic sarcoma of the distal femur that extended into the proximal 1/3 of the diaphysis and required a 28-cm resection. (**B**) A radiograph demonstrates how the surrounding bone responds to the compliant force over time.

All reconstructions at our institution were performed by the authors (JHH, CDM, EAA, PJB). The procedure followed the manufacturer’s recommended technique and has been described elsewhere [21]. Briefly, it entailed a sequence of steps after tumor or bone resection. The medullary canal was reamed just enough to accept the smallest anchor plug diameter of 12 mm or until there was endosteal contact for wider medullary canals. The anchor plug and traction bar were inserted into the canal. The muscle was bluntly split proximally to gain access to drill the bone, rather than disrupting the periosteal blood supply by stripping the bone. Using the outrigger for orientation (Fig. 1), three holes were drilled sequentially through the bone and anchor plug. After each hole was drilled, the drill bit was left in place to transfix both cortices and the anchor plug. After all three holes were drilled, the drill bits were replaced with fixation pins, which were tapped into place. We obtained a fluoroscopic image to confirm appropriate pin placement and length. Next, we used the conical reamer to prepare the surface of the host bone, constantly irrigating to prevent burning the bone and maintaining the periosteum as much as possible. The appropriate spindle size (small or large) and compressive force (400–800 pounds [181–363 kg]) varied according to the bone size and cortical thickness; compressive force levels recommended by the manufacturer were used (400 pounds [181 kg] for cortices 2.5–4.0 mm, 600 pounds [272 kg] for those 4.0–5.4 mm, 800 pounds [363 kg] for those ≥ 5.5 mm). The spindle and sleeve were placed over the intramedullary traction bar. The compression nut was tightened, approximately one half-turn beyond the point that initial resistance was felt, to compress the Belleville washers within the implant’s compression chamber. Although the manufacturer does not precisely specify the amount of torque required, the audible squeak of the washers signals that an appropriate level of tightening has been achieved (Video 1; supplemental materials are available with the online version of CORR). The remainder of the segmental knee arthroplasty was assembled as for the Orthopaedic Salvage System (OSS™) implant. In all patients, we used standard components, including an overall 8-cm anchor plug-traction bar construct; we did not use recently available options such as the 5-cm intramedullary implant. A typical case is illustrated, in which the implant was indicated for a short resection at the proximal femoral level (Fig. 2).

All patients underwent a similar rehabilitation regimen. Continuous passive motion was initiated on Postoperative Day 2 if there was no visible evidence of wound necrosis and continued for approximately 2 weeks for approximately 18 hours/day. Patients started walking on the first postoperative day using toe-touch weightbearing for 6 weeks, 50% weightbearing for an additional 6 weeks, and then progressive weightbearing as tolerated. All were fully weightbearing within a month. Patient adherence to these guidelines was presumed. However, at least one patient did not comply with recommended weightbearing proscription and sustained a periprosthetic fracture and implant failure after carrying a boat, necessitating revision. Chemotherapy was resumed 2 to 3 weeks postoperatively when appropriate for a patient’s diagnosis.

Patients were seen on a variable schedule based on the patient’s diagnosis and disease activity. For high-grade cancers, this was initially every 2 months and ultimately once per year after a 4-year disease-free interval. Patients with benign disease were seen every 3 to 6 months initially and then annually after 4 years. Standard AP and lateral radiographs were obtained at each visit. Radiographs were examined for any deformation of the implant that suggested bending or breaking of the device or fracture of the bone. Any new or increased pain was noted. Radiographic signs of loosening were not specifically quantified because, to our knowledge, there is no suitable methodology for evaluating loosening for this construct. Compress^®^ fixation failure was defined as revision of the fixation mechanism (anchor plug, traction bar, spindle, sleeve, or fixation pin) for any reason. Revision of intraarticular components (eg, polyethylene tibial bearing) that did not affect the bony fixation was not counted as a fixation or implant failure. Symptoms and signs of periprosthetic bone failure were noted and typically manifested as patient complaints of thigh pain and tenderness at the spindle-bone junction or reports of thigh pain during examination when the hip was rotated in the 90–90 position (supine, hip flexed 90°, knee flexed 90°). In such instances, radiographs were examined for evidence of a lack of osteointegration (ie, lack of hypertrophy in the bony segment between the spindle and the anchor plug and the presence of radiolucent lines at the spindle-bone interface). The presence of implant, bone, or symptomatic worsening prompted surgery.

In three patients who underwent revision surgery, we analyzed the bone adjacent to the junction site, both by visual inspection of the interface during surgery and by microscopic analysis of standard hematoxylin and eosin staining of decalcified samples.

Prosthesis-independent complications occurred but seemingly at a rate similar to what we have observed for other joint megaprostheses. There were three local recurrences; two were managed by local excision that did not affect the prosthesis and one required an amputation that removed the intact fixation. There were five prosthetic infections, including four primary infections and one secondary infection. The primary infections were successfully treated with washout, a change of intercalary segments, and retention of the Compress^®^ fixation; there were no recurrences of infection. The secondary infection necessitated amputation that included removal of the intact prosthesis. The intact implants removed by the two amputations were considered censored at the time of the removal.

We performed survival analysis of the device by the Kaplan-Meier log-rank technique using SPSS^®^ Version 14.0 (SPSS Inc, Chicago, IL, USA). Survival was defined as the time from the date of surgical implantation to the date of prosthesis removal or latest followup.

A comprehensive review of the literature was performed to place our results in context. A total of 718 articles were systematically read and reviewed. These were selected by performing a PubMed search on July 14, 2012, using the following key words: “megaprosthesis”, “femoral prosthesis”, “knee replacement”, and “tumor”. Articles that reported on the results of at least 20 patients for a mean of 5 years’ followup were considered. The results were further stratified based on studies that specified the number of distal femoral resections/reconstruction, the diagnoses for the surgical indications, and the prosthetic survivorship.

## Results

**Fig. 3:**
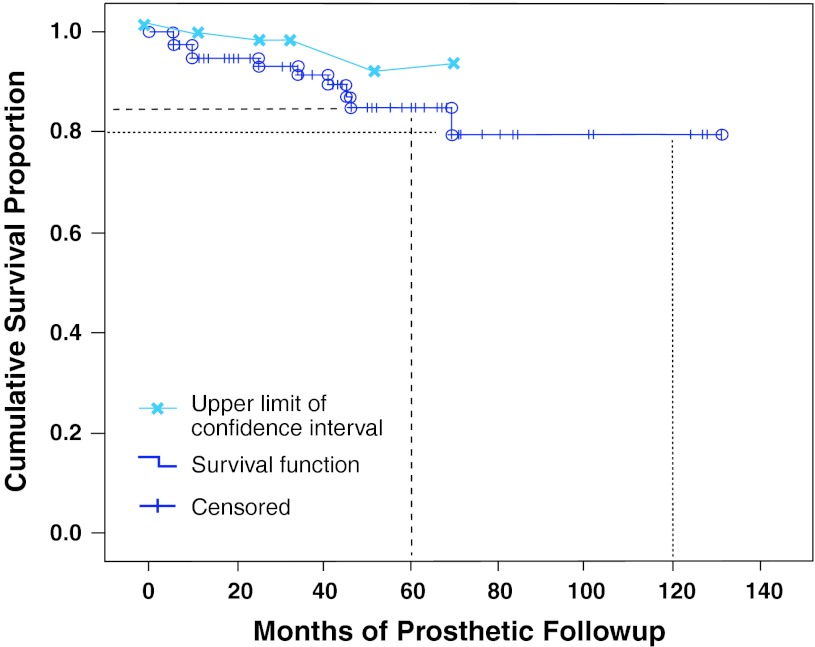
The Kaplan-Meier survival analysis of the Compress^®^ prosthesis shows implant survivorship is 85% at 5 years (dashed line) and 80% at 10 years (dotted line).

Survivorship of the Compress^®^ fixation was 85% at 5 years and 80% at 10 years (Fig. 3). There were three fixation failures in the first 2 years and five thereafter. Failures occurred throughout the followup period. Nevertheless, only one failure occurred among the 28 patients with more than 5 years of followup.

**Fig. 4:**
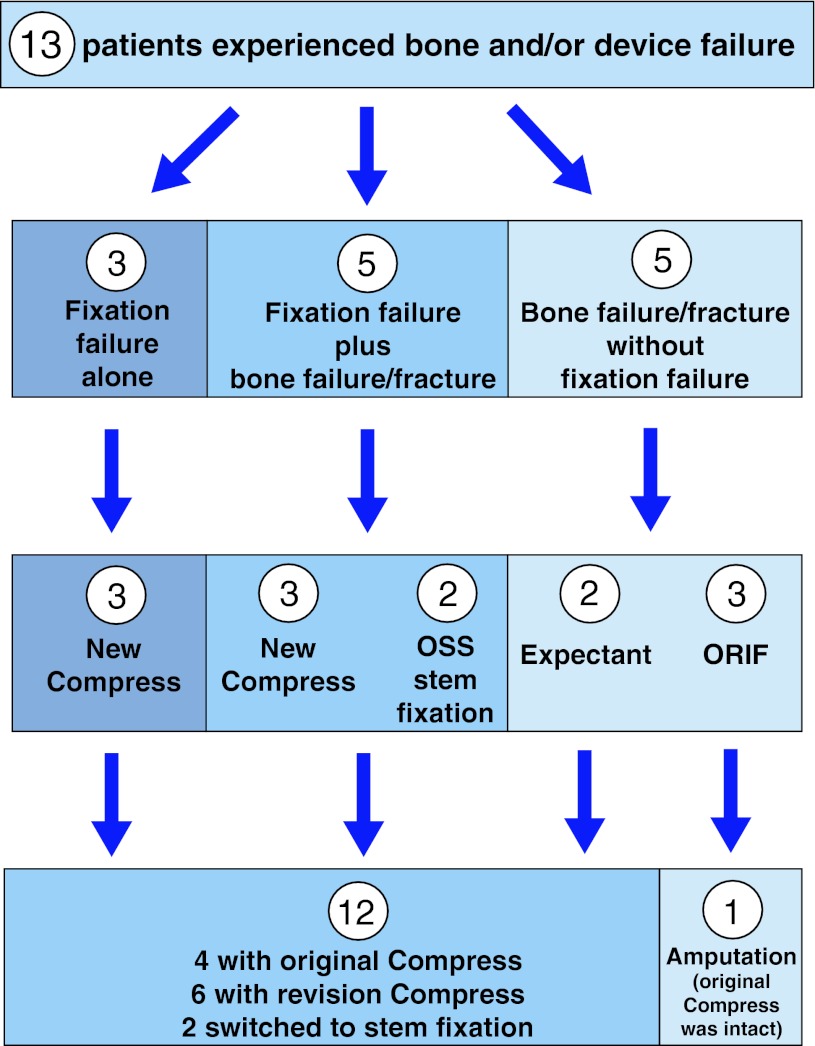
The flowchart shows the failure modes, treatment, and outcomes of the 13 patients who experienced bone and/or device failure after Compress^®^ implantation. ORIF = open reduction and internal fixation.

The modes of prosthetic failure that required revision surgery varied (Fig. 4). There were eight failures of the interface, due to aseptic loosening alone (three implants) or aseptic loosening with periprosthetic fractures that affected the interface (five implants).

**Fig. 5A–C:**
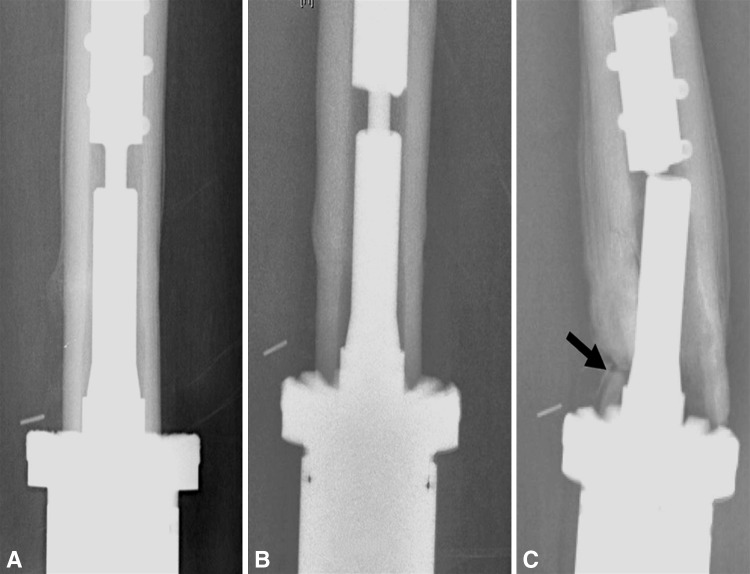
Type I failure is a combination of interface and bone failure. At (**A**) 3 and (**B**) 6 months, the bone has not integrated at the prosthetic interface, and (**C**) at 10 months, it has fractured between the spindle and the anchor plug (arrow). Notably, the sleeve and tension bar acted like an inadequate stem and the traction bar broke.

**Fig. 6A–B:**
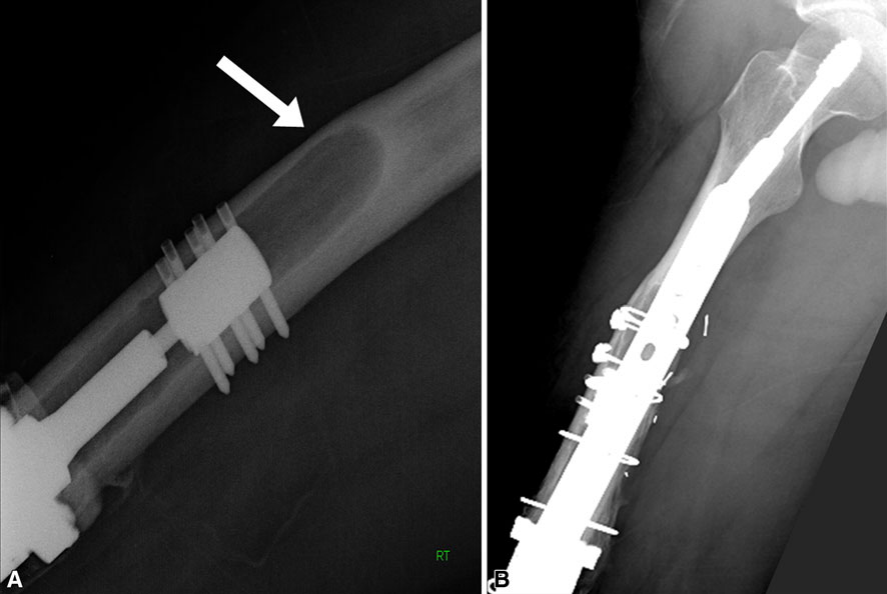
Type IIA failures are fractures proximal to the anchor plug fixation, perhaps at a stress transition point or, as in (**A**) this case where the bone was thin due to endosteal erosion from a failed prior stemmed implant (arrow). (**B**) The followup radiograph shows the fracture healed with a dynamic hip screw fixation and onlay allograft struts.

**Fig. 7A–B:**
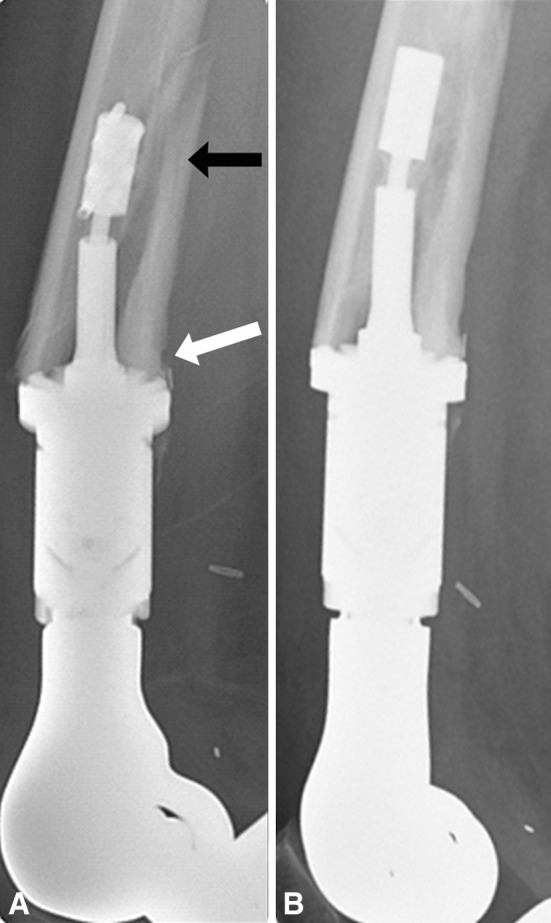
(**A**,** B**) Lateral radiographs taken 3 months apart show a Type IIB failure. (**A**) The black arrow points to the fracture of the posterior cortical segment that has moved with the spindle and the megaprosthesis, and the white arrow points to the intact bone-spindle interface where there has been some bone hypertrophy. Notably, the anterior cortex has not integrated and there is no hypertrophy. (**B**) The fracture healed spontaneously.

Ten patients had periprosthetic bone failure. Five of the eight patients with aseptic loosening had bone failure, characterized by the absence of bone growth into the porous spindle, collapse of the bone prosthesis interface, and associated fracture between the anchor pins and the spindle. As described above, revision was necessary. We classified this mode of bone failure as Type I: affecting the interface (Fig. 5). A second type of bone failure did not require prosthetic revision and had two subtypes. The first, Type IIA, included three fractures proximal to the implant that did not disrupt the fixation (Fig. 6). These fractures were treated and healed without any disruption of the prosthetic-bone interface. Two patients had fractures that healed uneventfully and retained their prosthesis with its original Compress^®^ fixation after further followup of 5 and 9 years, respectively. One patient healed her fracture but had an amputation for resultant osteomyelitis. Type IIB bone failure, which did not disrupt the interface or extend proximal to the anchor plug fixation pins, exhibited a unique pattern (Fig. 7). The spindle showed ingrowth at the posterior, but not anterior, aspect of the femur-spindle interface. Fracture occurred in a coronal plane. The integrated portion of the spindle remained attached to the posterior bone that fractured off as a segmental piece between the spindle and the anchor plug. There was some anterior angulation of the fracture, associated with a bent or broken traction bar. The anterior bone remained intact but had not integrated into the device, and a small separation was visible radiographically between anterior bone and the spindle. The displacement was not enough to require reduction. One patient from our cohort of 82 patients and the additional patient who underwent distal femoral reconstruction elsewhere had Type IIB failures. The first patient underwent an open bone-grafting procedure, at which time the fracture had already healed spontaneously. Because the second patient exhibited the same pattern, the fracture was allowed to heal without surgery, using only a functional fracture cast brace.

**Fig. 8A–C:**
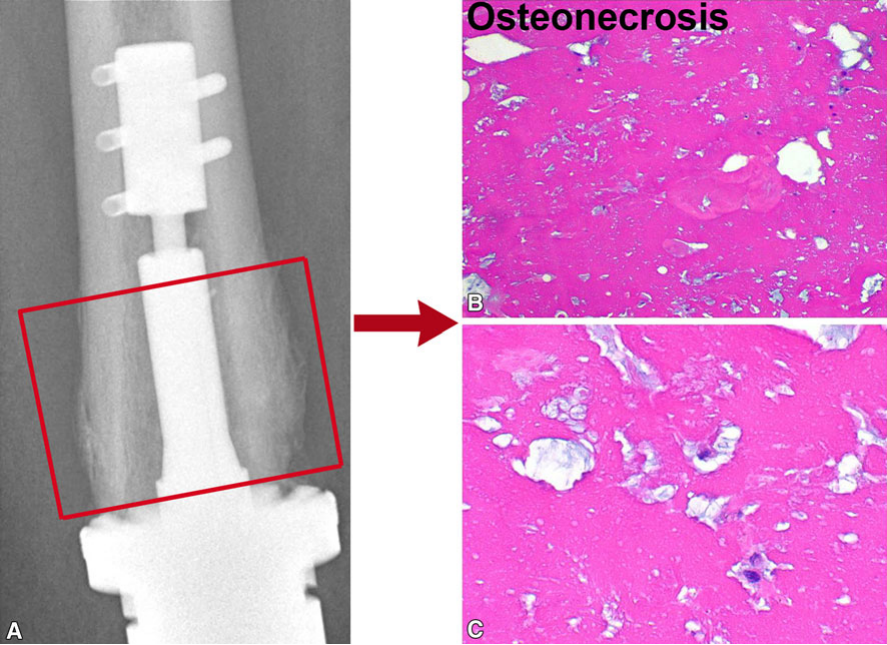
Type IIB failure shows (**A**) atrophy radiographically and (**B**,** C**) osteonecrosis histologically in two patient samples (Stain, hematoxylin and eosin; original magnification, ×40).

We performed a retrieval analysis of tissue in three patients from whom additional bone was removed, and there was no infection or cancer recurrence. Clinically, each patient had pain and radiographic failure. No well-fixed implants were analyzed. The specimens showed extensive osteonecrosis at the bone adjacent to the interface in each of the three specimens studied to date (Fig. 8). No other diagnostic histologic abnormalities were present.

## Discussion

Compliant, self-adjusting compression technology is a novel approach for durable prosthetic fixation of the knee. Early results have been encouraging, but longer followup reports are required from different centers. We therefore determined the survival of the Compress^®^ prosthesis at 5 and 10 actuarial years and identified the fracture-associated failure modes for this novel form of prosthetic fixation.

Several factors limit the interpretation of this study. First, the absence of a control group makes it impossible to compare results directly with fixation from conventional cemented or uncemented intramedullary stems. This study does not prove this fixation method is better than historic options, even though the authors believe this is generally true. Previously, we had hoped to address this question through a prospective study protocol that would have compared Compress^®^ fixation with press-fit and cemented stems. The protocol was proposed to a musculoskeletal oncology society, but the option of randomizing patients to the Compress^®^ was rejected by the society’s surgeons; hence, definitive comparative conclusions may never be possible. However, in this study, we noted all 18 patients who were revised to a Compress^®^ explicitly stated they were more comfortable with this implant, suggesting it was more stable and well fixed. Second, we only used the compliant fixation with a single design of rotating-hinge knee arthroplasty. Webber and Seidel [32] recently reported combining compliant fixation with a different body and articular design for pediatric limb salvage. Although the results are unlikely to have been different if a different knee design had been used, this question cannot be answered by this study. Patients in our cohort were treated over the course of a decade, and unrecognized differences in the population or surgical technique could have occurred. This problem plagues reviews of all low-incidence conditions that require many years to accumulate enough cases for analysis. Third, the minimum followup was set at only 1 year to allow inclusion of the two Type IIB cases of periprosthetic bone failure. The Kaplan-Meier method and survival curves allow the reader to see the time course of fixation failures and how the duration of followup may affect the prosthetic survival.

We found the survivorship of Compress^®^ implants for distal femoral reconstruction remained high, as confirmed in nearly twice as many patients as we previously reported and with followup extending to as long as 13 years. Eighty percent of patients retained their prosthesis after an actuarial 10 years of followup. These results extend our earlier report of 41 patients with a mean followup of 45 months (range, 3–97 months) [9]. The durability exceeds the survival of distal femoral/TKAs reported in most series, as reviewed by Bhangu et al. [4] and summarized in our systematic review (Table 3). Since 80% of patients with osteogenic sarcoma are currently expected to survive 10 years, prosthetic survival has yet to exceed patient survival. While the Compress^®^ implant performs at least as well over time as prostheses with other forms of bone fixation, further improvement of prosthetic durability is needed, especially for young patients with good long-term prognosis from their cancer. This high rate of prosthetic survival is encouraging but also suggests there may be factors regarding the patients, the surgery, or the subsequent management that influence the durability of the prosthesis. Notably, this analysis may be site specific and may not apply to compliant compression fixation in other sites [22] or under different clinical circumstances [2]. The time course of failure sheds some light on the nature of the fixation and its durability. Contrary to our previous report, on more than one occasion, the implant failed after the first year. Failures continued throughout the followup period. Indeed, five needed revision more than 2 years after implantation. However, only one of 28 implanted for more than 5 years subsequently failed. Thus, the concept that this form of biologic fixation would be long-lasting if it had a chance to become established was neither proven nor disproven in this cohort. It highlights how the durability is time dependent and the results can apparently be different depending on the time frame of the analysis.

**Table 3:** Summary of peer-reviewed literature reporting megaprosthesis survivorship

Study	Year	Number of patients	Site	Prosthesis*	Implant survival (%)	Followup (years)
Unwin et al. [31]	1993	218	Distal femur	Stanmore	65	10
Langlais et al. [15]	2006	26	Distal femur	GUEPAR^®^ II or custom press-fit cemented	92	12.5
Myers et al. [20]	2007	332	Distal femur	Stanmore	67	10
Zimel et al. [33]	2009	47	Distal femur	Howmedica 39 OSS™ 8	36	10
Farfalli et al. [9]	2009	50	Distal femur	OSS™ uncemented	71	10
Shehadeh et al. [27]	2010	101	Distal femur	MSRS™	70	10
Bergin et al. [3]	2012	93	Distal femur	MRS™/GMRS™	73	10
Tan et al. [28]	2012	78	Distal femur	Custom	71	10
Roberts et al. [26]	1991	135	Distal femur	Stanmore	72	5
Horowitz et al. [11]	1993	61	Distal femur	Burstein-Lane^®^	78	5
Kawai et al. [12]	1999	25	Distal femur	Finn^®^	88	5
Griffin et al. [10]	2005	74	Distal femur	KMFTR^™^ uncemented	70	14
Bruns et al. [8]	2007	13	Distal femur	MUTARS^®^	87	7
Kinkel et al. [13]	2010	49	Distal femur	MUTARS^®^	57	5
Matsumine et al. [17]	2011	69	Distal femur	Kyocera	85	5
Ritschl et al. [25]	1992	206	Mixed	KMFTR™	73	10
Unwin et al. [31]	1993	218	Mixed	Stanmore	65	10
Unwin et al. [30]	1996	1001	Mixed	Stanmore	67.4	10
Mascard et al. [16]	1998	90	Mixed	GUEPAR^®^	60	10
Mittermayer et al. [18]	2001	41	Mixed	KMFTR™	53	11
Plötz et al. [24]	2002	64	Mixed	Custom	25	10
Bickels et al. [6]^†^	2002	110	Mixed	MSRS™	88	10
Biau et al. [5]	2006	56	Mixed	Custom	50	11
Morgan et al. [19]	2006	105	Mixed	HMRS™	59	10
Current study	2012	82	Distal femur	Compress^®^	80	10

* Prostheses include Stanmore (Stanmore Implants Worldwide Ltd, Elstree, UK); GUEPAR^®^ II (Stryker France, Lyon, France); OSS™ = Orthopaedic Salvage System (Biomet Inc, Warsaw, IN, USA); MSRS™ = Modular Segmental Reconstruction System (Stryker Howmedica, Mahwah, NJ, USA); MRS™/GMRS™ = Modular Replacement System/Global Modular Replacement System stems (Stryker Howmedica); Burstein-Lane^®^ implant (Biomet Inc, Warsaw, IN, USA); Finn^®^ (Biomet Inc); KMFTR™ = Kotz Modular Femur Tibia Reconstruction System (Howmedica, Rutherford, NJ, USA); MUTARS^®^ = Modular Universal Tumour And Revision System (Implantcast GmbH, Buxtehude, Germany); Kyocera (Kyocera Medical Corp, Osaka, Japan); HMRS™ = Howmedica Modular Replacement System (Howmedica); ^†^patients in this study were also included in the analysis by Shehadeh et al. [27].

The unique fixation method of this prosthesis showed a unique spectrum of failure mechanisms. Aseptic loosening, commonly reported with other cemented and uncemented prostheses, also occurred with this implant. However, the aseptic loosening differed from that seen with other implants since bone ingrowth failed despite the continuously adjusting compression generated by the Belleville washers in the compression chamber. Retrieval specimens of these failures showed avascular necrosis of the underlying bone, in distinction to the viable bone found in well-fixed implants that were explanted for other reasons such as infection or tumor recurrence [7, 14]. A second, perhaps related unique finding was fracture or crumbling of the underlying bone between the anchor plug and the spindle. This was present in one patient who was included as part of a multicenter report on periprosthetic fractures around Compress^®^ devices [29]. The phenomenon has not been singled out for analysis. It could not be determined whether the osteonecrosis led to fatigue failure of the bone or the fracture caused osteonecrosis near the interface. The pathophysiology of these failures is unproven. Treatment of fractures related to prosthetic failure was not the focus of this study but is reportedly relatively easy and yields pain-free, functional reconstructions with few complications [1, 29].

Our analysis demonstrates a survivorship of 80% for Compress^®^ knee arthroplasty; the only published report demonstrating better survivorship after 10 years is that of Langlais et al. [15], who utilized custom-made press-fit femoral revision stems in 20 of the 26 joint arthroplasties. Thus, this report is the most comprehensive to date on an FDA-approved device for this unique form of prosthetic fixation.

## Electronic supplementary material

Below is the link to the electronic supplementary material.

Supplementary material 1 (MPG 20372 kb)
